# Feasibility of preoperative and postoperative physical rehabilitation for cardiac surgery patients – a longitudinal cohort study

**DOI:** 10.1186/s13102-023-00786-1

**Published:** 2023-12-19

**Authors:** Sandra Dijkstra, Johanneke Hartog, Joke Fleer, Pim van der Harst, Lucas H.V. van der Woude, Massimo A. Mariani

**Affiliations:** 1 Department of Cardio-Thoracic Surgery, University of Groningen, University Medical Center Groningen, Hanzeplein 1, 9713 GZ Groningen, The Netherlands; 2 Department of Health Sciences, University of Groningen, University Medical Center Groningen, Hanzeplein 1, 9713 GZ Groningen, The Netherlands; 3 Department of Cardiology, University of Groningen, University Medical Center Groningen, Hanzeplein 1, 9713 GZ Groningen, The Netherlands; 4 Department of Cardiology, University Medical Center Utrecht, Heidelberglaan 100, 3584 CX Utrecht, The Netherlands; 5 Center for Human Movement Sciences, University of Groningen, University Medical Center Groningen, Hanzeplein 1, 9713 GZ Groningen, The Netherlands; 6 Department of Rehabilitation Medicine, University of Groningen, University Medical Center Groningen, Hanzeplein 1, 9713 GZ Groningen, The Netherlands

**Keywords:** Cardiac surgical procedures, Preoperative exercise, Therapeutics, Observational study, Ergometry, Endurance training, Coronary artery Disease

## Abstract

**Background:**

This study aimed to determine the feasibility of a preoperative and postoperative (in- and outpatient) physical rehabilitation program, the Heart-ROCQ-pilot program.

**Methods:**

This cohort study included patients undergoing cardiac surgery (including coronary artery bypass graft surgery, valve surgery, aortic surgery, or combinations of these surgeries) and participated in the Heart-ROCQ-pilot program. Feasibility involved compliance and characteristics of bicycle and strength training sessions in the three rehabilitation phases.

**Results:**

Of the eligible patients, 56% (n = 74) participated in the program (41% of exclusions were due to various health reasons). On average across the rehabilitation phases, the compliance rates of bicycle and strength training were 88% and 83%, respectively. Workload to heart rate (W/HR) ratio and total absolute volume load for bicycle and strength training, respectively, improved in each rehabilitation phase (P < 0.05). The W/HR-ratio was higher during the last postoperative session compared to the first preoperative session (0.48 to 0.63 W/beat, P < 0.001) and similar to the last preoperative session (0.65 to 0.64 W/beat, P < 0.497). During less than 1% of the bicycle sessions, patients reported discomfort scores of 5 to 6 (scale 0–10, with higher scores indicating a higher level).

**Conclusions:**

The Heart-ROCQ-pilot program was feasible for patients awaiting cardiac surgery. Patients were very compliant and were able to safely increase the training load before surgery and regained this improvement within eight weeks after surgery.

**Supplementary Information:**

The online version contains supplementary material available at 10.1186/s13102-023-00786-1.

## Background

Many cardiac surgery patients are in poor physical condition before surgery, and this may affect postoperative surgical outcomes [1–3]. Unfortunately, elective patients experience further declines in physical, social, and psychological functioning during the waiting period for cardiac surgery [4, 5]. A longer waiting period has therefore been assumed to increase the risk of postoperative adverse events and mortality [4, 6]. In addition to the (high) impact of the surgery, patients experience further declines in physical functioning due to inactivity as well as bed rest during hospitalization after surgery [7, 8].

Preoperative rehabilitation (prehabilitation) with total body exercises has shown promising effects for the recovery after surgery in both cardiac and non-cardiac patient groups (e.g. improved physical capacity and shorter hospital stay) [9]. However, in cardiac surgery there have only been a limited number of prehabilitation studies, mainly focusing on inspiratory muscle training [10]. Little is known about the feasibility of prehabilitation in this patient group including total body exercises such as bicycle or strength training. As such, there are currently no practice guidelines for exercise training in the preoperative phase [11, 12].

Postoperatively, early (inpatient) rehabilitation has been shown to be safe, and to improve exercise capacity and quality of life [13–16]. However, the current guidelines are also limited for early postoperative inpatient rehabilitation. In contrast, postoperative outpatient rehabilitation is well studied and clearly defined in the guidelines, and therefore part of usual care in the Netherlands. Although clear evidence is available for the efficacy of postoperative outpatient rehabilitation (e.g., reduction in morbidity and mortality), many cardiac surgery patients are not enrolled (40%) in this type of rehabilitation [11, 17]. It is likely that enrolment in postoperative outpatient rehabilitation will increase when prehabilitation is offered [5].

Moreover, the combination of preoperative, early postoperative inpatient, and postoperative outpatient cardiac rehabilitation may speed up recovery from surgery, prevent adverse events, and prevent further progression of cardiovascular disease. The Heart-ROCQ-pilot program is one of the first programs with this combination of cardiac rehabilitation. Unlike previous prehabilitation [5, 18–20] or early postoperative (inpatient) programs [13–15] for cardiac surgery patients, the program combines both bicycle and strength training during pre- and postoperative (early in- and outpatient) rehabilitation. Furthermore, the program is multidisciplinary, including guidance from a physical therapist, dietician, and psychologist, which has been suggested to be an effective approach to promote cardiac health and optimize surgical outcomes [21, 22].

Studies evaluating preoperative or early postoperative inpatient rehabilitation mainly focus on the effects of a certain protocol, but often do not reflect on the actual capabilities or performance of the patients while executing that protocol. The actual frequency, intensity, and duration of total body exercises are very important for improving physical capacity. Though, it is still unclear what is feasible for this patient group, this is especially true since they may have a greater risk of sudden cardiac death during exercise [12]. Thus, the knowledge gap is that information about the feasibility, including the executed training characteristics and compliance, of a preoperative and postoperative program with total body exercises is scarce, while this may have a great impact on the efficacy of such a program [5, 11–15, 18–20, 23].

The aim of this study was therefore to evaluate the feasibility of the Heart-ROCQ-pilot program. Feasibility was assessed through evaluating the compliance and the characteristics of bicycle and strength training sessions during the Heart-ROCQ-pilot program. We hypothesized that patients awaiting cardiac surgery were able to safely exercise pre- and postoperatively and improve the actual bicycle and strength training characteristics (i.e., duration and intensity) during the Heart-ROCQ-pilot program.

## Methods

### Patients

Data were analysed of a cohort of all consecutive patients (≥ 18 years) scheduled for elective open heart surgery, including coronary artery bypass graft surgery, valve surgery, aortic surgery, or combinations of these surgeries in the University Medical Center Groningen (UMCG), the Netherlands, between March 2015 and August 2016 and who were invited to participate in the Heart-ROCQ-pilot program. Patients from regional hospitals are referred to the Heart Center of Groningen for Heart Team consultation immediately after diagnosis. This process normally takes 2–3 days. After the Heart Team makes a decision about the treatment, the referring cardiologist is notified the same day and the patient is placed on the waiting list within one week for surgery at the UMCG and concomitantly for inclusion in the Heart-ROCQ-pilot program. Patients were excluded if they had comorbidities that prevented participation in one of the program elements (e.g., disorders to the nervous or musculoskeletal system that limit exercise capacity, severe COPD with GOLD classification ≥ 3, non-coping behaviour, addiction, serious psychological illness) or if exercise was deemed undesirable (i.e., hypertrophic cardiomyopathy, unstable angina, advice from a cardiologist). Furthermore, patients were excluded if they were unable to understand or read Dutch instructions. Patients who did not give permission to use data or who followed an adapted program (i.e., not able to follow bicycle training) were also excluded from the analysis. Data were collected from existing databases and medical records. All collected data were anonymised in according with the Dutch Privacy Law. Ethical approval and consent for the study were waived by the Medical Ethics Board of the UMCG. Since this was a feasibility study, no sample size calculation was done.

### Heart-ROCQ-pilot program

**Fig. 1:**
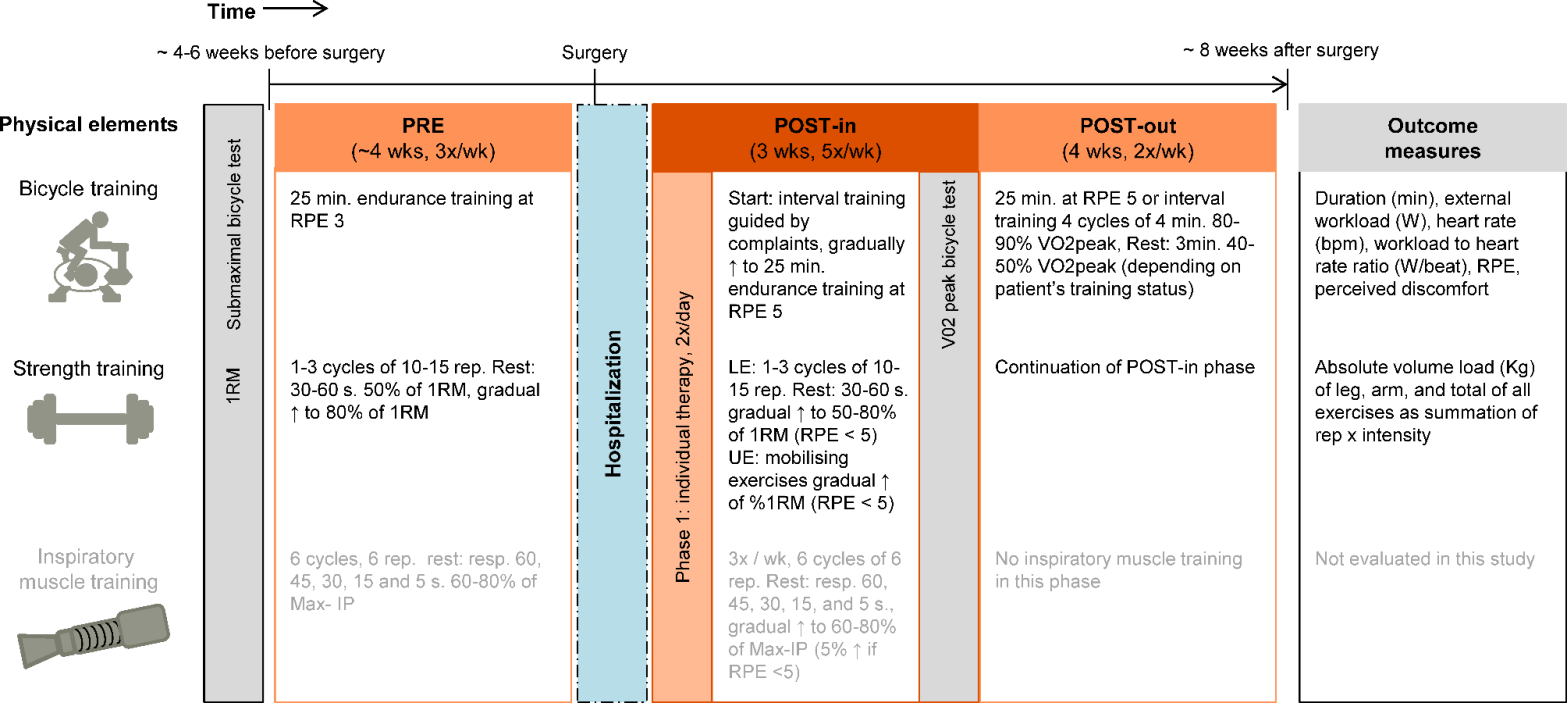
Overview of the physical elements of the Heart-ROCQ-pilot program. ↑: An increase; LE: lower extremities; Max-IP: Maximum inspiratory pressure; rep: repetitions; RM: repetition maximum; RPE: rate of perceived exertion (Borg scale 0–10); UE: upper extremities; VO2: oxygen uptake

The Heart-ROCQ-pilot program consists of three phases: preoperative outpatient (PRE phase: minimum of 4 weeks until surgery, 3x/wk.), early postoperative inpatient (POST-in phase: 3 weeks, 5x/wk. during a working week), and postoperative outpatient (POST-out phase: 4 weeks, 2x/wk.) rehabilitation (Fig. 1). Key elements of the program were bicycle and strength training (Fig. 1). In order to estimate the individual, initial intensity of bicycle and strength training, different physical tests were performed [12]. Preoperatively, a submaximal bicycle test and six to ten repetition maximum (RM) of six strength exercises (triceps dips, rowing, chest press, seated leg press, seated leg curl, leg extension) were conducted (Fig. 1). The submaximal bicycle test used a 5–25 W/min ramp protocol and was ended when 70% of expected heart rate or expected workload was reached, or standard indications to terminate a test occurred [12]. Postoperatively, at the end of the POST-in phase, a symptom limited ergometer test with a 5–25 W/min ramp protocol and cardiopulmonary measurements was performed (Fig. 1). Expected workload was calculated based on gender, age, and height. The value of the expected workload defined the height of the steps of the ramp protocol. After establishing the initial intensity during the first bicycle training session, training sessions were based on the rate of perceived exertion (RPE; Fig. 1). All bicycle and strength sessions were conducted under the supervision of two physical therapists specialized in cardiac rehabilitation. During bicycle training patients were monitored with continuous 3-wire lead ECG. Additional elements of this multidisciplinary program were swimming, sports and games, inspiratory muscle training, and guidance from a dietician, a psychologist, an occupational health consultant, and a stop-smoking consultant. These elements were not evaluated in the current study.

### Feasibility

#### Compliance

Compliance (%) was defined as the number of actual bicycle and strength training sessions completed by the patients relative to the number of offered sessions. The offered sessions were those planned and presented to the patients. The sessions according to the protocol were as prespecified in the protocol (Fig. 1). It was assumed that patients completed a session when data of the training equipment was available. When malfunctioning of the equipment occurred (i.e., no absence was recorded in the patient rehabilitation planning systems, but no data was available for any of the scheduled patients for that session), the patient was assumed to be present during that session. The reasons for not offering a session according to the protocol and a patient’s absence were also evaluated.

### Training characteristics

The training characteristics for bicycle training were duration (min), external workload (W), heart rate (bpm), workload to heart rate ratio (W/HR-ratio [W/beat]), and a subjective evaluation (Fig. 1). Subjective evaluation was assessed on the Borg scale 0–10, with higher scores indicating a higher level of the three components: RPE of fatigue and shortness of breath, and perceived discomfort [24]. The isopower ergometers with ECG (Corival IV Rehab and Lode ECG streamer, Lode) monitored (5 Hz) the external workload and the heart rate. For strength training, the absolute volume load (Kg) of leg (VL-leg, i.e., seated leg press, seated leg curl, and leg extension), arm (VL-arm, i.e., triceps dips, rowing, and chest press), and total of all exercises (VL-total) were evaluated (Fig. 1). Equipment for strength training (Enraf-Nonius) monitored the training characteristics in sets, repetitions, and intensity.

### Data analyses

Data of bicycle training was exported using the LCRM software (v2.15.0, Lode). MATLAB (vR2018a, Mathworks) was used to interpolate and filter (8th order low-pass Butterworth, cut-off frequency: 0.1 Hz) the heart rate data. Subsequently, the mean values of external workload and heart rate of the training phase (excluding warm-up and cooling down) over each session were calculated. W/HR-ratio was calculated by dividing the mean external workload by the mean heart rate of the training phase over each session. Data of strength training was exported from the En-Track software (v6.29.4, Enraf-Nonius EN-track). Per session, the VL-arm, VL-leg, and VL-total (Formula 1) were calculated using STATA statistical software (v15.0, StataCorp).

Formula 1:

$${\sum\nolimits_{iExercise}^{nExercise} {(\sum\nolimits_{iSet}^{nSet} {Repetitions} } _{iSet}} \times \,Intensity_{_{iSet}}^{}{)_{iExercise}}$$

With iExercise is the number of the exercise and iSet is the number of sets of the exercise [25].

### Statistical analyses

Statistical analyses were performed using IBM SPSS statistics software (v23). Descriptive statistics were used to present patient characteristics, compliance, RPE, and perceived discomfort. Repeated measures analyses were performed to analyse differences in training characteristics for each rehabilitation phase separately, using external workload, heart rate, W/HR-ratio, VL-leg, VL-arm, and VL-total as dependent variables. The within subject factor was the first and last session of each phase. The analyses were repeated with the last session in the POST-out phase and the first and last sessions in the PRE phase as the within subject factor. Patients were excluded from the analysis of a phase due to missing data when data from only one session in a phase was available or when there were only two sessions available either at the beginning or at the end of a phase. When the data consisted of different sample sizes, separate repeated measures analyses (or paired samples t-tests) were performed. Non-parametric equivalents were performed when the assumption of normal distribution was not met. All statistical tests were two-sided and P-values < 0.05 were considered statistically significant.

## Results

### Patients

**Fig. 2:**
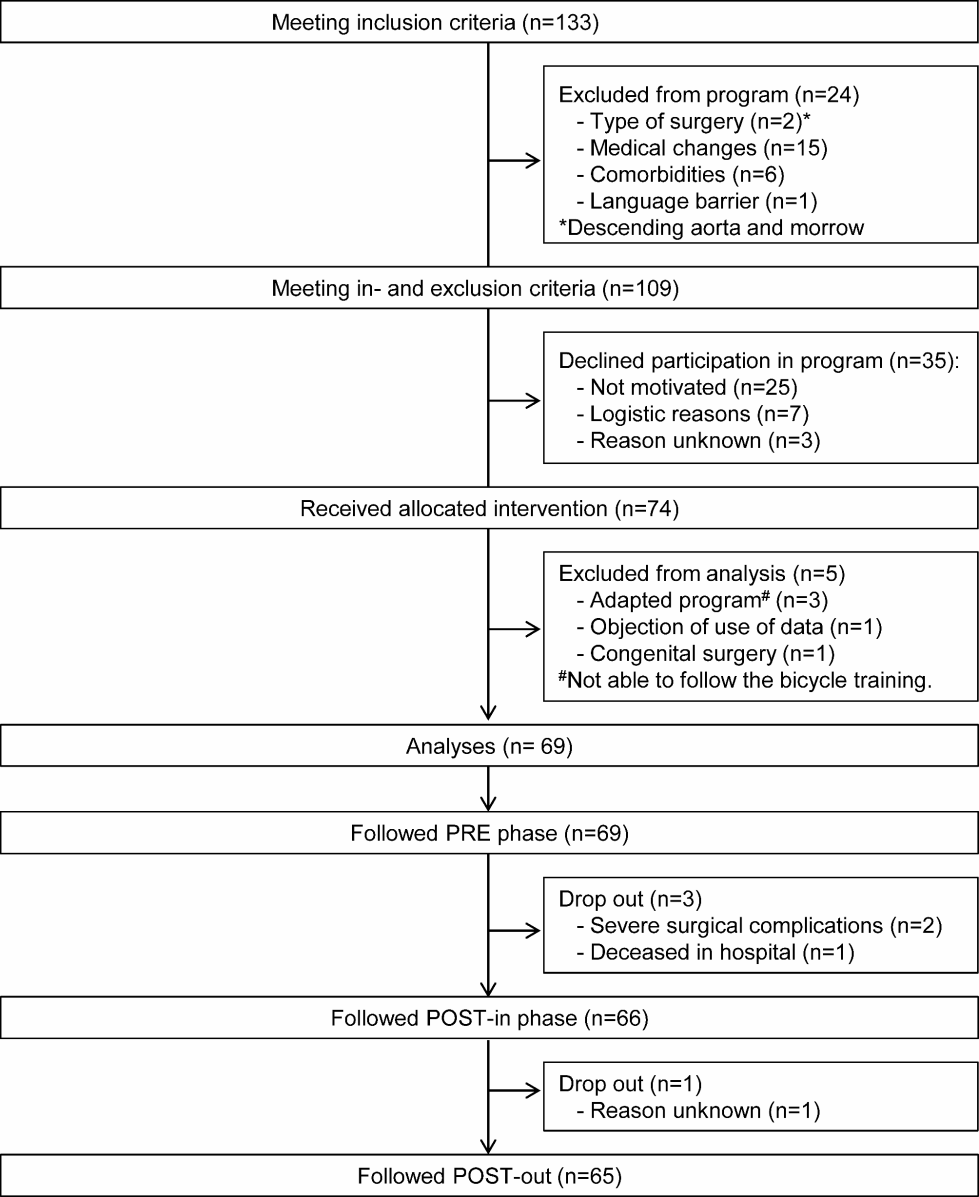
Consort flow diagram of patients in- and excluded in the Heart-ROCQ-pilot program and drop-outs during the three rehabilitation phases

Of the eligible patients, 56% (n = 74) participated in the Heart-ROCQ-pilot program (Fig. 2). Of those excluded, 41% (n = 24) did not meet the exclusion criteria and 59% (n = 35) declined participation mostly due to lack of motivation (n = 25). A further five patients were excluded from the analyses, because the program needed to be adapted (n = 3), they objected to the use of data (n = 1) or they underwent congenital surgery (n = 1). The characteristics of the 69 analysed patients are shown in Table 1. Of the included patients, 58% of the surgeries were an isolated CABG and 17% were another surgical procedure than a CABG. Of the remaining proportion, the surgeries were a combination of two interventions (22% of the patients) or three interventions (3% of the patients), which could also be a combination including a CABG. Only six patients underwent an isolated mitral valve surgery, and as institutional policy, isolated mitral valve surgery is performed via minimally invasive right anterolateral thoracotomy at our department.

**Table 1:** Patient and in-hospital characteristics

Patient preoperative characteristics	Heart-ROCQ-pilot program (n = 69)
Age (years)	65 ± 9.0
Male	53 (77)
Body mass index (Kg/m^2^)	27.7 ± 5.0
Left ventricular function (LVF)	
Poor LVF (< 31%)	2 (3)
Moderate LVF (31–50%)	20 (29)
Good LVF (> 50%)	47 (68)
NYHA class Class I Class II Class III Class IV	4 (6) 45 (65) 18 (26) 2 (3)
Atrial fibrillation/flutter	13 (19)
Diabetes mellitus	16 (23)
Chronic lung disease^a^	5 (7)
Recent myocardial infarction	6 (9)
Previous percutaneous coronary intervention	19 (28)
Previous cardiac surgery	3 (4)
History of stroke	4 (6)
logistic EuroSCORE II	1.3 (0.9–2.2)
**In-hospital characteristics**	
Type of surgery CABG isolated Single, non-CABG - Valve procedure - Aorta surgery Two interventions Three interventions	40 (58) 12 (17) 11 (92) 1 (8) 15 (22) 2 (3)
On-pump surgery	37 (54)
Bypass time (min)	160.3 ± 87.0
Cross clamp time (min)	105.4 ± 69.7
Surgery time (min)	237.1 ± 93.9
Prolonged mechanical ventilation > 24u	5 (7)
Prolonged ICU stay^b^	11 (16)
Re-admission ICU	5 (7)
Hospital stay (days, median (IQR))	6 (6–10)
Hospital mortality	1 (1)

Values expressed as mean ± SD or n (% yes), unless otherwise noted. CABG: Coronary artery bypass graft; ICU: Intensive care unit

^a^Long-term use of bronchodilators or steroids for lung disease

^b^Two or more calendar days from ICU admission to discharge

### Feasibility

#### Compliance

**Fig. 3:**
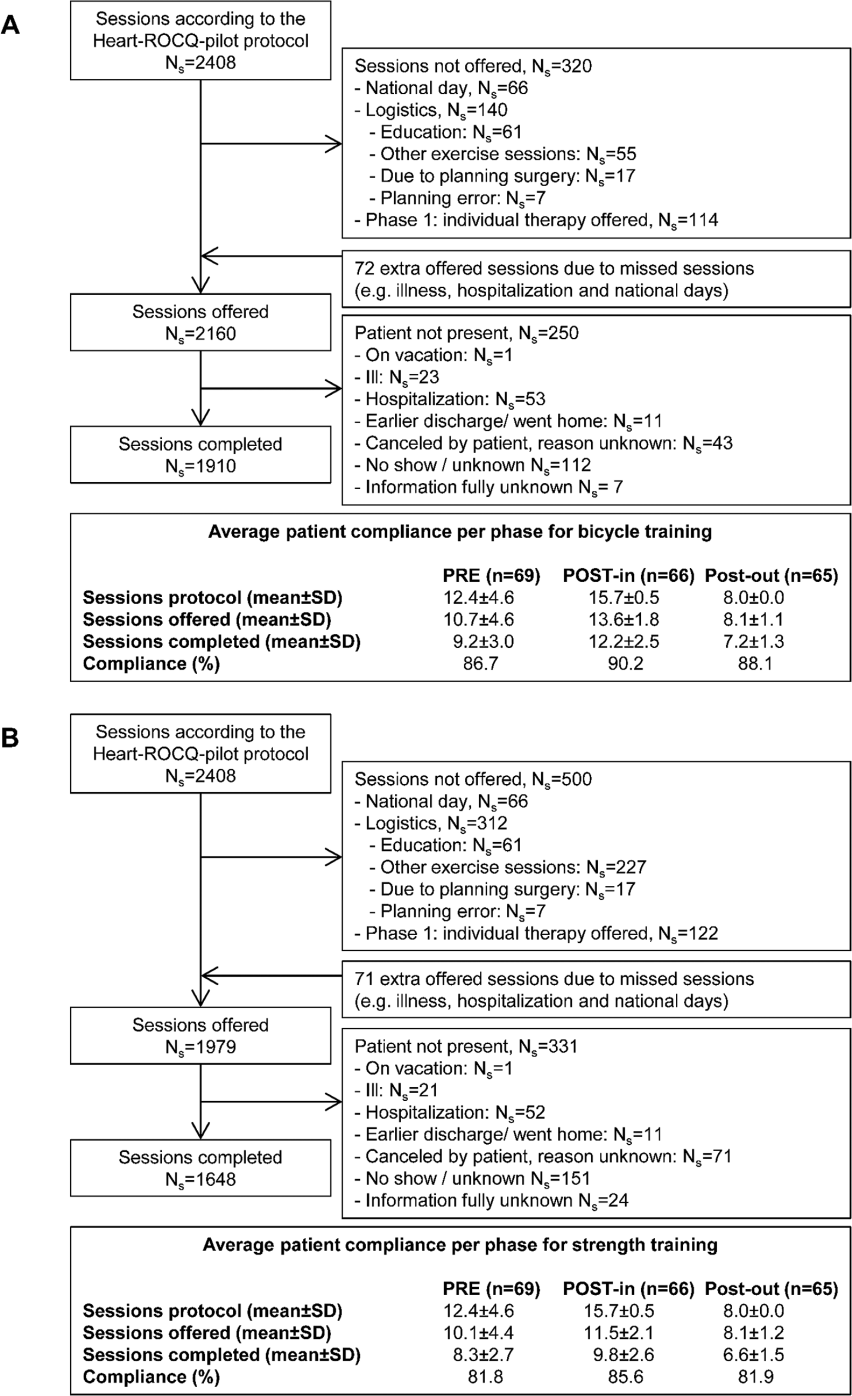
Number of bicycle (**A**) and strength (**B**) training sessions in the three rehabilitation phases according to the protocol, offered to and actually completed by the patients. n: number of patients; Ns: number of sessions

Figure 2 also shows the number of patients included in the analyses of the three rehabilitation phases and the reasons for drop out. Figure 3 shows the number of bicycle and strength training sessions in each rehabilitation phase and the reasons why a session was not planned or completed. All 69 patients completed on average 9 and 8 sessions of respectively bicycle and strength training in the PRE phase, which represented a compliance rate of respectively 86.7% and 81.8%. During this phase, one patient was admitted to the hospital due to an infected wound seroma. The surgery of two patients changed from elective to a more urgent basis because of unstable angina pectoris. One of these patients participated for 2.5 weeks in the PRE phase and for the other patient the duration of the PRE phase was 11 weeks (prolonged because of a consultation with a urologist). Postoperatively, eight patients (12%) were readmitted to the hospital (median: 4 days, IQR: 1–8 days), of whom two were readmitted twice. The reasons for readmissions were arrhythmias, dyspnoea, fever, superficial sternal wound infection, and collapsing. After readmissions all patients were able to continue in the same but extended phase (n = 7) or the subsequent phase (n = 1). The compliance rates of the postoperative rehabilitation phases for both bicycle and strength training were also above 80% (Fig. 3). On average across the rehabilitation phases, patients completed 88% and 83% of the offered sessions of bicycle and strength training, respectively.

### Training characteristics

The median duration of the PRE phase from the initial bicycle test was 38 days (IQR: 37–42) during a waiting time with a median duration of 55 days (IQR: 50–66) (from the day of acceptance to surgery). At the start of the POST-in phase, patients received individual therapy for a median of 2 days (IQR: 1–2) with a maximum of 6 days. Patients started at a median of 8 days (IQR: 7–12) after surgery with group sessions of bicycle and strength training. Details about the physical tests to determine the individual, initial training intensity are shown in S1 Table (see Additional file 1).

**Fig. 4:**
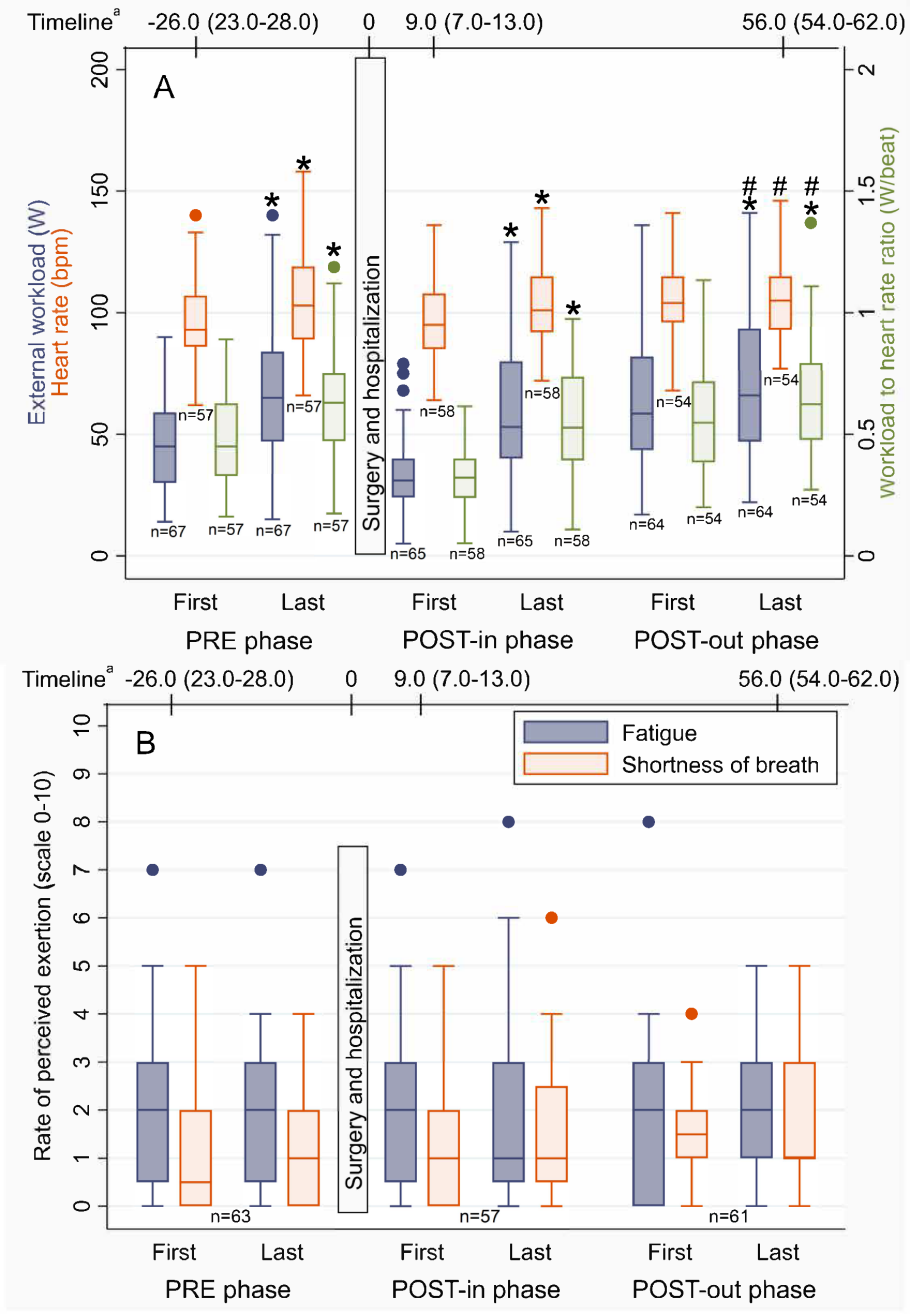
Training load of bicycle training at the first and last session of the three rehabilitation phases. (**A**) External workload, heart rate, and workload to heart rate ratio; (**B**) Rate of perceived exertion. Differences paired samples t-tests: *first vs. last session in a rehabilitation phase; #first session of PRE phase vs. last session of POST-out phase; +last session of PRE phase vs. last session of POST-out phase. ^a^ days (Median (IQR)) relative to the surgery

The results of the bicycle training are shown in Fig. 4 and S2 Table (see Additional file 2). In the PRE- and POST-in phases, external workload, heart rate, and W/HR-ratio significantly increased (P < 0.05). In the POST-out phase, external workload increased (P < 0.001), while heart rate remained the same (P = 0.642), which was reflected in an increase in the W/HR-ratio (P < 0.001). These three outcome measures were significantly higher during the last postoperative session compared to the first preoperative session (p < 0.001), but similar to the last preoperative session (P > 0.05). Figure 4B shows that the median for RPE of fatigue and shortness of breath was below three in the first and last session in each rehabilitation phase. Patients reported a perceived discomfort score of 0 (scale 0–10, with higher scores indicating a higher level of discomfort) during the majority of all bicycle sessions in the three rehabilitation phases (range of 80–86% across the phases), a discomfort score of 0.5 to 2 in 11–16% of the sessions, and in 3–5% of the sessions a discomfort score of 3 to 4 was reported. In less than 1% of the sessions a score of 5 to 6 for discomfort was reported. The highest level of discomfort was seven, which was reported once during the PRE phase due to pain between the shoulder blades. In 6% of the sessions, no score of perceived discomfort was reported at all.

The median durations of the first and last session of bicycle training of all phases were 25 min (excluding warm-up and cooling down), except for the first session of the POST-in phase which had a median of 21 min (IQR: 14–25). Because of knee complaints, one patient switched to another ergometric device and was therefore excluded from these analyses. Another patient was excluded from the PRE phase analysis, because only one session was available.

**Fig. 5:**
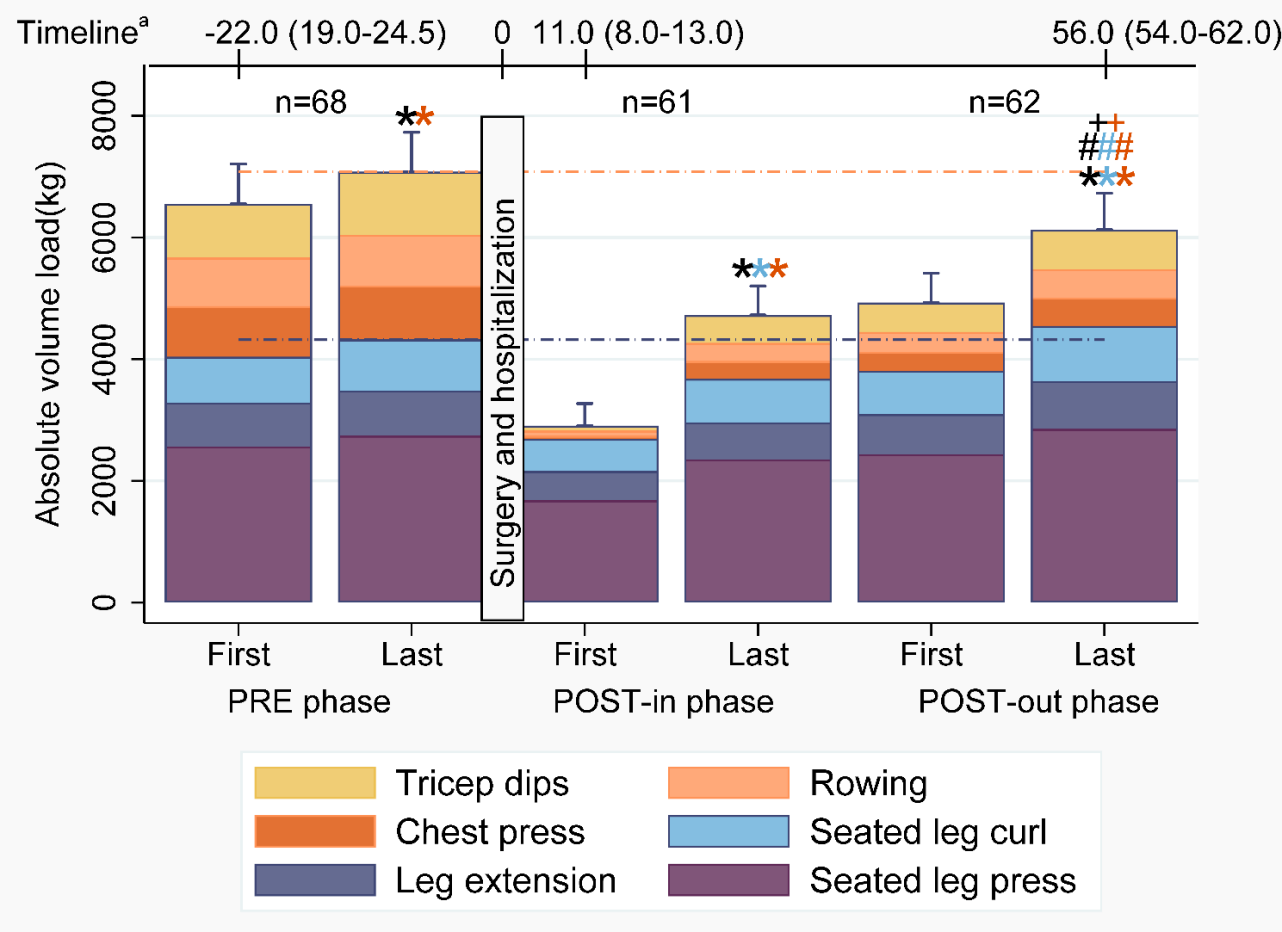
Absolute volume load (sets*repetitions*intensity) of the strength training at the first and last session of the three rehabilitation phases. Mean with upper limit of 95% confidence interval are shown. Differences of repeated measures analyses of all exercises (black), leg exercises (blue), and the arm exercises (orange): *first vs. last session in a rehabilitation phase; #first session of PRE phase vs. last session of POST-out phase; +last session of PRE phase vs. last session of POST-out phase. a days (Median (IQR)) relative to the surgery

Strength training results are shown in Fig. 5 and S3 Table (see Additional file 3) and show significant increases in VL-leg, VL-arm, and VL-total in each rehabilitation phase (P < 0.05), except for VL-leg in the PRE phase (P = 0.124). The VL-leg during the last postoperative session was significantly higher than the first preoperative session (P = 0.033) and was comparable with the last preoperative session (P = 0.658). For VL-arm and VL-total the last postoperative session was significantly lower than the first and last preoperative session (P < 0.05). At the start of the POST-in phase, patients performed the arm exercises with a median of 2 days (IQR: 0–3) later than the leg exercises. In total, nine patients (PRE: n = 1, POST-in: n = 5, POST-out: n = 3) were excluded from the analysis of that particular phase since only two sessions either at the beginning or at the end of a phase or just one session was available.

Due to the introduction of newly developed exercise equipment some additional missing values were present in the first and last session of a training phase. Therefore, in 12% of the sessions the adjacent session was chosen for analysis. In 2% of the cases there was no external workload available in the first, last, or adjacent session, thus the first or last session from which data was available was used. Heart rate data was missing in 31 sessions since it was not completely recorded (n = 5) or a high variability was present with no indication of atrial fibrillation (n = 26). All analyses of external workload and W/HR-ratio showed similar statistical results when patients with atrial fibrillation were excluded.

## Discussion

The purpose of this study was to determine the feasibility of the Heart-ROCQ-pilot program with preoperative and postoperative (in- and outpatient) physical rehabilitation in elective cardiac surgery patients. The results showed that 56% of eligible patients participated in the program. Patients completed on average 88% and 83% of the offered sessions of bicycle and strength training, respectively. In each phase the external workload and W/HR-ratio during bicycle training increased significantly. The same applied to the VL-total during strength training. During the majority of the sessions (80–86% in the three rehabilitation phases) no discomforts (score 0) were reported and patients rarely (< 1% of the sessions) reported discomfort scores of 5 to 6 (scale 0–10, with higher scores indicating a higher level of discomfort).

The sample of this study was representative with respect to patient characteristics compared to the Dutch population undergoing cardiac surgery [26]. Furthermore, the enrolment in the Heart-ROCQ-pilot program was comparable to usual care cardiac rehabilitation in the Netherlands [17] and the United States [27], and other prehabilitation studies [5, 18, 19, 23]. Remarkably, patients included in the Heart-ROCQ-pilot program underwent higher-risk surgeries as well (seen as a wide range in EuroSCORE), compared to previous prehabilitation studies [5, 18, 19]. Despite the additional types of sessions due to the multidisciplinary nature and intensive postoperative inpatient program when compared to other prehabilitation programs, patients were very compliant to the Heart-ROCQ-pilot program [5, 18, 23]. These findings suggest that this program is well suitable for “all-comers” undergoing cardiac surgery.

To our knowledge, this is the first study that present detailed information about the training characteristics and trainability of patients before and early after cardiac surgery. Evaluating training characteristics provided insights into the capabilities and performance of patients during physical cardiac rehabilitation. This is paramount for the efficacy of any such cardiac rehabilitation program. The results of the training characteristics of the bicycle sessions (increases in W/HR-ratio and workload in each of the three rehabilitation phases, but without changes in heart rate in the POST-out phase and RPE values in all three phases) indicate better cardiac functioning and thereby improved exercise tolerance. Furthermore, these findings suggest that patients listed for cardiac surgery are able to improve their training load. Within eight weeks after surgery, patients improved their training load during bicycle training and leg strength exercises compared to the first session preoperatively. Patients were, however, not able to recover to their preoperative load regarding arm exercises. This is probably because of the healing process of the sternotomy in the first six to eight weeks after surgery, where patients trained at lower frequencies and intensities to accommodate [11, 12]. Bicycle and strength training, especially leg exercises, were thus feasible components of the Heart-ROCQ-pilot program.

Since cardiac surgery patients may have a greater risk of sudden cardiac death during exercise, safety is very important [12]. To ensure safety, several measures have been included in the Heart-ROCQ-pilot program. In close collaboration with the supervising cardiologists, only patients without unstable angina or progressions in complaints were referred to the Heart-ROCQ-pilot program. Moreover, all patients underwent bicycle ergometry preoperatively to obtain information on their capability to exercise. In addition, all bicycle sessions were conducted under the supervision of two physical therapists specialized in cardiac rehabilitation and with continuous ECG monitoring. The majority of the patients reported no perceived discomfort and patients rarely reported a score of 5 or higher for perceived discomfort. A previous study suggested that patients participated safely in the preoperative part of the Heart-ROCQ-pilot program and were not at higher risk for postoperative complications, while it might even prevent postoperative atrial fibrillation [28]. Thus, despite their heart disease, our selection of patients were able to safely exercise in the Heart-ROCQ-pilot program prior to and early after cardiac surgery.

This feasibility study was initiated to acquire more insight into the trainability of patients listed for cardiac surgery as a preparation for the design of the Heart-ROCQ PROBE study [29]. Little is known about the trainability of patients before (and early) after cardiac surgery, because prehabilitation studies mainly focused on inspiratory muscle training [10] and measurement of maximal exercise capacity is complex in this patient group due to potential medical contra-indications and occurrence of kinesiophobia [12]. The strength of this study is that we circumvented these aspects by evaluating the actual performed training load (W) in combination with how the body responded physiologically (heart rate) and the patient’s subjective experience (RPE and perceived discomfort). Patients experienced the strain of bicycle training lower than expected (Fig. 4, RPE medians < 3). Changing the stop criteria of the preoperative submaximal ergometry test from 70 to 90% of the expected maximal heart rate will possibly improve the tailoring of the initial training load to the individual. Nevertheless, the external workload during bicycle training in the preoperative phase and at the end of the early postoperative inpatient phase was comparable to those seen in two other studies that showed an improvement in quality of life and a reduced hospital stay in their patient group [5, 13]. Moreover, improvements in training characteristics were shown with the current exercise intensity. The Heart-ROCQ-pilot program could, therefore, possibly reduce physical and psychological risk factors and work as a means of secondary prevention as well by promoting a healthy lifestyle. Our currently ongoing randomized controlled trial, the Heart-ROCQ PROBE study, will show whether these improvements in training characteristics will lead to an enhanced recovery after cardiac surgery [29]. The Heart-ROCQ PROBE study determines the effects of this program on physical and psychological functioning, as well as on the cost-effectiveness and lifestyle risk factors, in comparison to postoperative cardiac rehabilitation provided in usual care [29].

This study had some limitations. When interpreting the results, it should be considered that the use of medication, such as beta-blockers, may have changed during the rehabilitation program. In addition, the presence of missing values could have influenced the results. Even though there is always a risk of interferences when using ECG, newly developed exercise equipment showed many interferences in the ECG signals at the start of this study. By improving this equipment, the number of interferences and thus missing values in heart rate during the study was reduced. Because we used a 3-wire ECG lead, it was not reliable to evaluate ECG changes during the bicycle sessions. A 3-wire ECG lead is, however, easier to use for patients during cardiac rehabilitation. Our study included a relatively small convenience sample of selected group of patients (44% of eligible patients was not included), which in itself impacts the study’s generalizability. Characteristics of the group of excluded patients are unknown, though they could possibly be less compliant, since 25 patients were excluded on the basis of a lack of motivation. Patients might be more motivated to participate in a home-based rehabilitation program, especially when rehabilitation centers are far away from where patients live. Therefore, future research should also investigate the feasibility of home-based rehabilitation programs for higher-risk patients as well, such as those awaiting cardiac surgery, while also providing proper monitoring in order to evaluate the trainability and to ensure safety [30]. Moreover, the effect of the program in different types of patients (e.g., in terms of gender or type of underlying disease and its consequent surgery) is unclear and this is deemed to be of interest for future research. The percentage of postoperative readmissions in this study (12% of the patients) was lower compared to earlier reported figures in cardiac surgery (15–25% of the patients) [31]. Since no control group was included, we could not investigate whether this was due to the selected patient group or a better recovery.

## Conclusions

The Heart-ROCQ-pilot program was feasible for patients awaiting cardiac surgery. Despite their heart disease, patients were very compliant and were able to safely increase the training load before surgery and regain it within eight weeks after surgery. More research is needed on this promising program to evaluate the effects of preoperative and postoperative physical rehabilitation on surgical outcomes, overall functioning, and health in order to further improve clinical care before and after cardiac surgery.

### Electronic supplementary material

Below is the link to the electronic supplementary material.

Supplementary S1 Table. Test outcomes used to determine the individual, initial training intensity of the Heart-ROCQ-pilot program

Supplementary S2 Table. Differences in the characteristics of external workload, heart rate and their ratio of bicycle training at the first and last session in the three rehabilitation phases

Supplementary S3 Table. Differences in the characteristics of the absolute volume load of strength training at the first and last session in the three rehabilitation phase

## Data Availability

All data generated or analysed during this study are included in this published article [and its supplementary information files].

## List of abbreviations

POST-in: Early postoperative inpatient rehabilitation
POST-out: Postoperative outpatient rehabilitation
PRE: Preoperative rehabilitation
RM: Repetition maximum
RPE: Rate of perceived exertion
UMCG: University Medical Center Groningen
VL-arm: Absolute volume load for arm exercises
VL-leg: Absolute volume load for leg exercises
VL-total: Absolute volume load for all exercises
W/HR-ratio: Workload to heart rate ratio
