# Supplementary material for: Feasibility of preoperative and postoperative physical rehabilitation for cardiac surgery patients – a longitudinal cohort study
Source: BMC Sports Sci Med Rehabil. 2023 Dec 19;15:173. doi: 10.1186/s13102-023-00786-1 (PMC10731823; doi:10.1186/s13102-023-00786-1)
Supplement: Supplementary file 1 — Supplementary S1 Table. Test outcomes used to determine the individual, initial training intensity of the Heart-ROCQ-pilot program [file 13102_2023_786_MOESM1_ESM.docx]

**S1 Table. Test outcomes used to determine the individual, initial training intensity of the Heart-ROCQ-pilot program**

| **Start of preoperative rehabilitation** | **Heart-ROCQ-pilot program** |
| --- | --- |
| Maximum workload preoperative bicycle test (W, n=67)^a^ | 90±28 |
| Maximum heart rate preoperative bicycle test (bpm, n=67)^a^ | 106±14 |
| 1RM seated leg press (Kg, n=63) | 113.7±35.4 |
| 1RM seated leg curl (Kg, n=64) | 33.9±10.6 |
| 1RM leg extension (Kg, n=63) | 32.9±10.7 |
| 1RM chest press (Kg, n=62) | 39.3±13.7 |
| 1RM triceps dips (Kg, n=57) | 47.8±16.7 |
| 1RM rowing (Kg, n=64) | 36.2±12.2 |
| **At the end of POST-in phase** |  |
| Maximum workload postoperative bicycle test (W, n=51) | 97±42 |
| Maximum heart rate postoperative bicycle test (bpm, n=51) | 115±21 |
| Maximum VO2 postoperative bicycle test (mL/min/kg, n=49) | 15.7±4.7 |

*Values expressed as mean ± standard deviation. ^a^The maximum value during the preoperative bicycle test, which was ended when 70% of expected heart rate or expected workload was reached, or standard indications to terminate a test occurred [12]. RM: repetition maximum; VO2: oxygen uptake.*
