# Supplementary material for: Feasibility of preoperative and postoperative physical rehabilitation for cardiac surgery patients – a longitudinal cohort study
Source: BMC Sports Sci Med Rehabil. 2023 Dec 19;15:173. doi: 10.1186/s13102-023-00786-1 (PMC10731823; doi:10.1186/s13102-023-00786-1)
Supplement: Supplementary file 2 — Supplementary S2 Table. Differences in the characteristics of external workload, heart rate and their ratio of bicycle training at the first and last session in the three rehabilitation phases [file 13102_2023_786_MOESM2_ESM.docx]

**S2 Table. Differences in the characteristics of external workload, heart rate and their ratio of bicycle training at the first and last session in the three rehabilitation phases.**

|  | **First session**  (mean±SD) | **Last session**  (mean±SD) | **t(df)-value** | **P-value** |
| --- | --- | --- | --- | --- |
| **External workload (W)** |  |  |  |  |
| PRE phase | 45±18 | 67±27 | *t*(66)= -11.329 | p<0.001 |
| POST-in phase | 32±15 | 59±27 | *t*(64)= -11.894 | p<0.001 |
| POST-out phase | 62±28 | 71±29 | *t*(63)= -7.586 | p<0.001 |
| First session PRE phase vs. last session POST-out phase | 46±17 | 70±29 | *t*(62)= -9.878 | p<0.001 |
| Last session PRE phase vs. last session POST-out phase | 69±27 | 70±29 | *t*(62)= -0.694 | p=0.490 |
| **Heart rate (bpm)** |  |  |  |  |
| PRE phase | 96±18 | 105±20 | *t*(56)= -5.066 | p<0.001 |
| POST-in phase | 97±16 | 104±17 | *t*(57)= -3.323 | p=0.002 |
| POST-out phase | 105±16 | 106±17 | *t*(53)= -0.467 | p=0.642 |
| First session PRE phase vs. last session POST-out phase | 95±17 | 106±17 | *t*(50)= -4.710 | p<0.001 |
| Last session PRE phase vs. last session POST-out phase | 103±18 | 105±17 | *t*(53)= -0.887 | p=0.379 |
| **Workload to heart rate ratio (W/beat)** | |  |  |  |
| PRE phase | 0.48±0.18 | 0.64±0.22 | *t*(56)= -11.878 | p<0.001 |
| POST-in phase | 0.32±0.12 | 0.54±0.21 | *t*(57)= -11.143 | p<0.001 |
| POST-out phase | 0.57±0.23 | 0.64±0.24 | *t*(53)= -7.076 | p<0.001 |
| First session PRE phase vs. last session POST-out phase | 0.48±0.18 | 0.63±0.21 | *t*(50)= -7.112 | p<0.001 |
| Last session PRE phase vs. last session POST-out phase | 0.65±0.23 | 0.64±0.23 | *t*(53)= 0.683 | p=0.497 |
