# Supplementary material for: Feasibility of preoperative and postoperative physical rehabilitation for cardiac surgery patients – a longitudinal cohort study
Source: BMC Sports Sci Med Rehabil. 2023 Dec 19;15:173. doi: 10.1186/s13102-023-00786-1 (PMC10731823; doi:10.1186/s13102-023-00786-1)
Supplement: Supplementary file 3 — Supplementary S3 Table. Differences in the characteristics of the absolute volume load of strength training at the first and last session in the three rehabilitation phase [file 13102_2023_786_MOESM3_ESM.docx]

**S3 Table. Differences in the characteristics of the absolute volume load of strength training at the first and last session in the three rehabilitation phase.**

|  | **First training**  (mean±SD) | **Last training**  (mean±SD) | **F(df,df) value** | **P-value** |
| --- | --- | --- | --- | --- |
| **Absolute volume load, leg exercises (kg)** | |  |  |  |
| PRE phase | 4038.7±1786.1 | 4322.7±1771.7 | F(1,67)=2.427 | p=0.124 |
| POST-in phase | 2690.0±1287.9 | 3678.6±1589.4 | F(1,60)=60.207 | p<0.001 |
| POST-out phase | 3809.1±1592.1 | 4546.8±1900.6 | F(1,61)=30.206 | p<0.001 |
| First session PRE phase vs. last session POST-out phase | 4160.4±1729.8 | 4555.3±1915.1 | F(1,60)=4.781 | p=0.033 |
| Last session PRE phase vs. last session POST-out phase | 4496.4±1656.3 | 4555.3±1915.1 | F(1,60)=0.199 | p=0.658 |
| **Absolute volume load, arm exercises (kg)** | | |  |  |
| PRE phase | 2514.3±1101.3 | 2758.6±1070.5 | F(1,67)=7.581 | p=0.008 |
| POST-in phase | 215.5±448.5 | 1046.1±404.3 | F(1,60)=115.557 | p<0.001 |
| POST-out phase | 1121.9±406.9 | 1580.2±624.8 | F(1,61)=66.374 | p<0.001 |
| First session PRE phase vs. last session POST-out phase | 2559.8±1066.6 | 1568.5±623.1 | F(1,60)=79.524 | p<0.001 |
| Last session PRE phase vs. last session POST-out phase | 2762.7±1078.1 | 1568.5±623.1 | F(1,60)=111.159 | p<0.001 |
| **Absolute volume load, total exercises (kg)** | | |  |  |
| PRE phase | 6552.9±2706.7 | 7081.3±2678.9 | F(1,67)=5.239 | p=0.025 |
| POST-in phase | 2905.5±1430.2 | 4724.7±1869.8 | F(1,60)=109.119 | p<0.001 |
| POST-out phase | 4931.0±1901.1 | 6127.0±2363.7 | F(1,61)=52.898 | p<0.001 |
| First session PRE phase vs. last session POST-out phase | 6720.2±2652.3 | 6123.8±2383.2 | F(1,60)=6.205 | p=0.016 |
| Last session PRE phase vs. last session POST-out phase | 7259.1±2595.9 | 6123.8±2383.2 | F(1,60)=37.218 | p<0.001 |
